# Development of Two-Dimensional Model of Photosynthesis in Plant Leaves and Analysis of Induction of Spatial Heterogeneity of CO_2_ Assimilation Rate under Action of Excess Light and Drought

**DOI:** 10.3390/plants11233285

**Published:** 2022-11-29

**Authors:** Ekaterina Sukhova, Daria Ratnitsyna, Ekaterina Gromova, Vladimir Sukhov

**Affiliations:** Department of Biophysics, N.I. Lobachevsky State University of Nizhny Novgorod, 603950 Nizhny Novgorod, Russia

**Keywords:** CO_2_ assimilation, excess light, spatial heterogeneity, leaf CO_2_ conductance, two-dimensional photosynthetic model, drought

## Abstract

Photosynthesis is a key process in plants that can be strongly affected by the actions of environmental stressors. The stressor-induced photosynthetic responses are based on numerous and interacted processes that can restrict their experimental investigation. The development of mathematical models of photosynthetic processes is an important way of investigating these responses. Our work was devoted to the development of a two-dimensional model of photosynthesis in plant leaves that was based on the Farquhar–von Caemmerer–Berry model of CO_2_ assimilation and descriptions of other processes including the stomatal and transmembrane CO_2_ fluxes, lateral CO_2_ and HCO_3_^−^ fluxes, transmembrane and lateral transport of H^+^ and K^+^, interaction of these ions with buffers in the apoplast and cytoplasm, light-dependent regulation of H^+^-ATPase in the plasma membrane, etc. Verification of the model showed that the simulated light dependences of the CO_2_ assimilation rate were similar to the experimental ones and dependences of the CO_2_ assimilation rate of an average leaf CO_2_ conductance were also similar to the experimental dependences. An analysis of the model showed that a spatial heterogeneity of the CO_2_ assimilation rate on a leaf surface should be stimulated under an increase in light intensity and a decrease in the stomatal CO_2_ conductance or quantity of the open stomata; this prediction was supported by the experimental verification. Results of the work can be the basis of the development of new methods of the remote sensing of the influence of abiotic stressors (at least, excess light and drought) on plants.

## 1. Introduction

Photosynthesis is a key process in the life of green plants and the basis of their productivity. It is a complex process [1,2] that can be strongly affected by numerous abiotic stressors, including excess light [3,4,5] and fluctuations in light intensity [6,7,8,9], drought [10,11,12], decrease [13] and increase [14,15,16] in temperatures, and others.

Changes in the photosynthetic processes induced by the action of stressors include both the damage of photosynthetic machinery and numerous protective responses. The stressor-induced damages include photodamage under excess light [3,4,5], increase in proton leakage across the thylakoid membrane under heating [14], damage of photosynthetic complexes through the stimulation of the production of reactive oxygen species induced by the decrease in photosynthetic dark reactions under the action of various stressors [17], and others. The protective responses include the induction of a non-photochemical quenching [3,4,18,19], stimulation of a cyclic electron flow around photosystem I [7,19,20], translocation of Ferredoxin-NADP^+^ Reductase [21,22], activation of photorespiration [23], changes in the positions of chloroplasts [24,25,26], and others. These processes can strongly interact, e.g., the stimulation of the cyclic electron flow increases the acidification of the lumen in chloroplasts and can increase an energy-dependent component of the non-photochemical quenching caused by this acidification [19,27,28] through the interacted protonation of PsbS proteins [3,4,29] and the synthesis of zeaxanthin and antheraxanthin from violaxanthin in the xanthophyll cycle [30].

The complexity of the photosynthetic stress responses is a reason for the active development of mathematical models of photosynthetic processes [31], because these models can be effective tools for the prediction of changes in photosynthesis under the action of adverse factors. There are models simulating processes on different levels of the organization of photosynthesis [31]: models of the ways of energy utilization in the reaction centers of photosystem II [32,33,34], models focusing on the description of photosynthetic light reactions and their regulation by stressors [5,35,36,37,38,39,40], models focusing on the description of photosynthetic dark reactions and CO_2_ fluxes [41,42,43,44], complex models of plant productivity [45,46], and global photosynthetic models [47,48].

The photosynthetic model by Farquhar, von Caemmerer, and Berry (FvCB model) [42,49,50,51] is a widely-used model of C_3_ photosynthesis that can describe the photosynthetic processes in mesophyll cells, leaves, plant canopies, and ecosystems [31]. This model is based on a stationary description of a photosynthetic CO_2_ assimilation rate (A_hv_) that is dependent on the slowest process of three processes that can limit the dark reactions of photosynthesis [50]: CO_2_ fixation by Rubisco, linear electron flow (LEF) in the electron transport chain of thylakoids, and triose flux from the stroma of chloroplasts. Particularly, the FvCB model can be used for the description of the heterogeneity of the photosynthetic processes in the leaves and canopies of plants [52,53,54,55,56]; analysis of this heterogeneity has great importance for revealing new factors that can regulate photosynthetic processes (e.g., the influence of changes in the intensity and spectrum of light caused by an increase in the distance from the leaf surface during photosynthetic processes or the influence of 3-D microstructures of leaf tissues and chloroplast movements on photosynthesis). 

However, the simulation of photosynthetic processes in the scale of a leaf surface that can also be based on the FvCB model is weakly developed. A model of photosynthetic processes in the scale of a leaf surface is a potential tool for the theoretical investigation of the spatial heterogeneity of photosynthetic parameters on this surface, including revealing possible modifications of the heterogeneity under the action of stressors. There are several reasons supporting the importance of the development of the leaf photosynthesis model and its theoretical analysis.

First, revealing stressor-induced changes in the photosynthetic heterogeneity can provide an additional indicator of the action of adverse factors on plants. It can be used for the development of new methods for remote sensing plant stress changes. Particularly, these methods can be based on the measurements of the spatial heterogeneity of the distribution of a photochemical reflectance index (PRI), which is calculated based on reflectance at 531 and 570 nm [57,58,59,60] and is strongly related to photosynthetic parameters (the non-photochemical quenching of fluorescence, effective quantum yield of photosystem II, light-use efficiency, and photosynthetic CO_2_ assimilation rate) [61,62,63,64,65,66,67].

Second, the development of the leaf photosynthesis model and revealing stressor-induced changes in the spatial photosynthetic heterogeneity can be an important step for further investigation into new mechanisms influencing plant tolerance to stressors. Particularly, it was theoretically shown that the spatial heterogeneity in the physiological parameters of two-dimensional models of living cells can modify their responses to the actions of external factors through a diversity-induced resonance [68,69,70], e.g., this effect was shown for excitable plant cells under cooling [70,71]. It cannot be excluded that the spatial heterogeneity in photosynthetic processes can also influence the plant response to stressors. Potentially, the leaf photosynthesis model can also be used as an analysis tool for this influence.

Thus, there were three main purposes of our work: (i) The development and verification of the two-dimensional model of C_3_ photosynthesis in the plant leaf, which was based on the FvCB model. (ii) The model-based analysis of the induction of the spatial heterogeneity of the CO_2_ assimilation rate under excess light conditions and a decrease in leaf CO_2_ conductance (g_S_) (this g_S_ decrease imitated the action of a short-term drought). (iii) Additional experimental verification of the results of this analysis.

## 2. Description of the Two-Dimensional Model of C_3_ Photosynthesis in Plant Leaves

**Figure 1:**
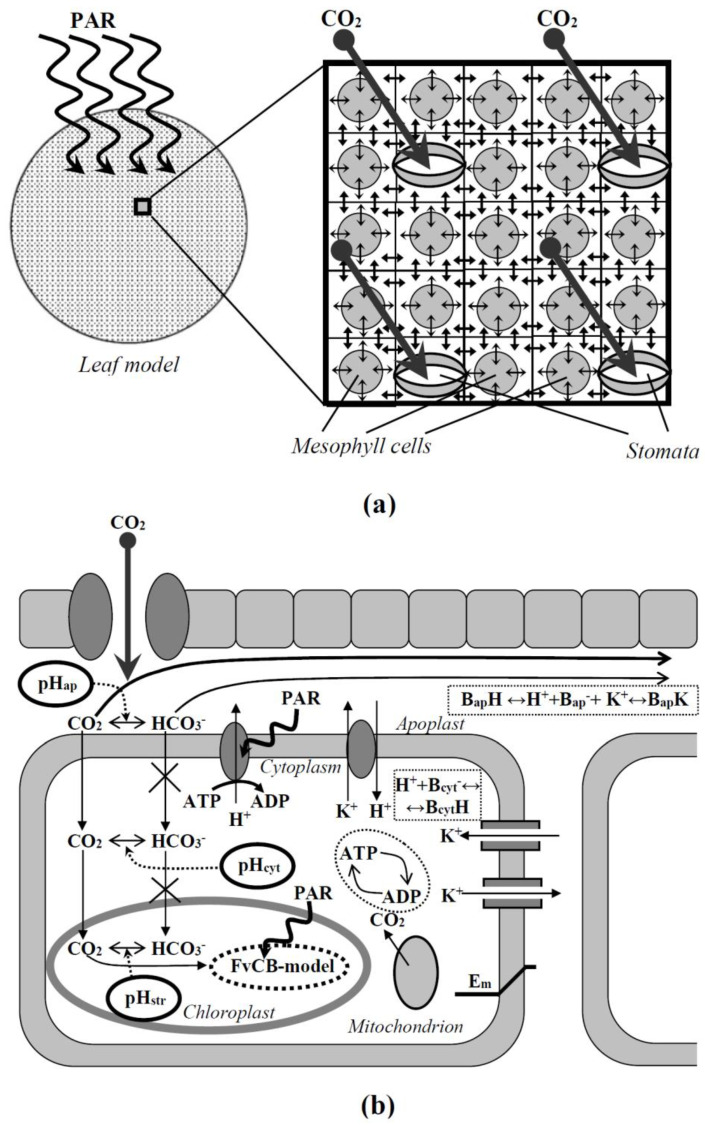
A general scheme of the developed two-dimensional model (**a**) and main processes described by the model on the cell level (**b**). Model elements (squares) include both mesophyll cells and stomata or only mesophyll cells (without stomata). Small arrows in the general scheme show transport of carbon dioxide, H^+^, and K^+^ between apoplastic volumes of neighboring cells and across the plasma membrane. PAR is the photosynthetic active radiation. pH_ap_, pH_cyt_, and pH_str_ are pH in the apoplast, cytoplasm, and stroma of chloroplasts, respectively. B_cyt_^−^ and B_cyt_H are the free and proton-bound cytoplasmic buffers. B_ap_^−^, B_ap_H, and B_ap_K are the free, proton-bound, and potassium-bound apoplastic buffers. E_m_ is the difference of electrical potentials across the plasma membrane. FvCB model is the Farquhar–von Caemmerer–Berry model. The main systems of ion transport at rest, including H^+^-ATP-ases, H^+^/K^+^-antiporters, inwardly rectifying K^+^ channels, and outwardly rectifying K^+^ channels, are described in the two-dimensional photosynthetic model.

The two-dimensional model of C_3_ photosynthesis in the plant leaf was based on the round system of elements (Figure 1a). Each element included descriptions of the photosynthetic cell and the apoplast; some elements (central elements in 3 × 3 elements squares or in 5 × 5 elements squares) additionally included stomata. Figure 1b shows the main processes considered in the model. Equations and parameters of the two-dimensional model of C_3_ photosynthesis in the plant leaf are described in File S1 “Equations and parameters of the two-dimensional photosynthetic model” in detail.

Briefly, the simplified FvCB model, which described only two limiting stages (the CO_2_ fixation by Rubisco and the linear electron flow in the electron transport chain of thylakoids in accordance with [51]), was used as the basis for the simulation of the photosynthetic CO_2_ assimilation in mesophyll cells (in accordance with standard Equation (1) [50,51]):

(1) $$A_{\mathrm{hv}}=\min\left(W_{c},{\;W}_{j}\right)\frac{{\left[{\mathrm{CO}}_{2}\right]}_{\mathrm{str}}-\Gamma^{*}}{{\left[{\mathrm{CO}}_{2}\right]}_{\mathrm{str}}}$$

where W_c_ and W_j_ are carboxylation rates at the Rubisco-limited CO_2_ assimilation and electron transport-limited CO_2_ assimilation conditions, respectively (both values were calculated based on standard Equations (S2) and (S3) in accordance with [50]), [CO_2_]_str_ is the concentration of CO_2_ in the stroma of chloroplasts, Γ^*^ is the photosynthetic CO_2_ compensation point in the absence of mitochondrial respiration. It should be noted that Equation (1) was used for the estimation of the measured photosynthetic CO_2_ assimilation and for comparison with the experimental results. The photosynthetic consumption of CO_2_ in the stroma was described as min(W_c_, W_j_); i.e., the correction relating to photorespiration was not used in this case. Photorespiration was separately described as the CO_2_ source in the cytoplasm in accordance with Equation (2) based on Equation (1):

(2) $$V_{\mathrm{phr}}=\frac{A_{\mathrm{hv}}\Gamma^{*}}{{\left[{\mathrm{CO}}_{2}\right]}_{\mathrm{str}}}$$

A dark respiration was described as another CO_2_ source in the cytoplasm. In accordance with von Caemmerer et al. [1], it was assumed that the rate of the dark respiration (R_d_) was constant.

Carbon fluxes between cells and compartments were described based on Fick’s law [72,73,74]. CO_2_ fluxes across the stomata (j_S_), plasma membrane (j_PM_), and envelopes of chloroplasts (j_Chl_), which depended on the CO_2_ conductance [74,75], were analyzed in the model (Equations (3)–(5)):

(3) $$j_{S}=g_{S}{}^{0}\left({\left[{\mathrm{CO}}_{2}\right]}_{\mathrm{out}}-{\left[{\mathrm{CO}}_{2}\right]}_{\mathrm{ap}}\right)$$

(4) $$j_{\mathrm{PM}}=g_{\mathrm{PM}}\left({\left[{\mathrm{CO}}_{2}\right]}_{\mathrm{ap}}-{\left[{\mathrm{CO}}_{2}\right]}_{\mathrm{cyt}}\right)$$

(5) $$j_{\mathrm{Chl}}=g_{\mathrm{Chl}}\left({\left[{\mathrm{CO}}_{2}\right]}_{\mathrm{cyt}}-{\left[{\mathrm{CO}}_{2}\right]}_{\mathrm{str}}\right)$$

where [CO_2_]_out_, [CO_2_]_ap_, and [CO_2_]_cyt_ are concentrations of CO_2_ in the air, apoplast and cytoplasm, respectively; g_S_^0^, g_PM_, and g_Chl_ are CO_2_ conductance for the stomata, plasma membrane, and chloroplast envelopes (j_Chl_), respectively.

Similar HCO_3_^−^ fluxes were assumed to be absent, because charged HCO_3_^−^ should weakly diffuse across biological membranes [75].

The lateral fluxes of both neutral CO_2_ and charged HCO_3_^−^ in the apoplast were considered in the model [73] and were described by Equations (S12) and (S13). In accordance with our previous work [70], it was assumed that each cell had its section of apoplast. The lateral fluxes were described between nearest sections (four lateral fluxes for each apoplast section, Figure 1a).

The ratios between the concentrations of CO_2_ and HCO_3_^−^ were dependent on pH in the apoplast, cytoplasm, and stroma of chloroplasts [75]. It was assumed that the transitions between CO_2_ and HCO_3_^−^ were fast; this meant that the stationary distribution between these molecules could be used. Equation (6) described the portion of CO_2_ in the total content of CO_2_ and HCO_3_^−^:

(6) $$P_{{\mathrm{CO}}_{2}}=\frac{1}{1+10^{\mathrm{pH}-\mathrm{pK}}}$$

where pK is the negative logarithm of the equilibrium constant in the reaction of the transition between CO_2_ and HCO_3_^−^.

The stromal pH was assumed to be constant; the pH in the apoplast and cytoplasm was described based on our early model of ion transport and electrogenesis in plant cells [76].

The description of H^+^ and K^+^ fluxes across the plasma membrane was based on our previous model [70,76], which was simplified. Only H^+^-ATPase, inwardly and outwardly rectifying K^+^ channels, and K^+^/H^+^-antiporters were described, because the interaction of these systems could support stationary H^+^ concentrations in the cytoplasm and the apoplast: the H^+^-ATPase provided the primary transport of H^+^ across the plasma membrane; the K^+^ channels provided the K^+^ transport, which electrically compensated the charge transfer related to the proton transport through H^+^-ATPase; the K^+^/H^+^-antiporter prevented the non-physiological increase in cytoplasmic pH and K^+^ concentration and the decrease in apoplastic pH and K^+^ concentration.

The buffer properties of the cytoplasm (for H^+^) (Equation (S37)) and the apoplast (for K^+^ and H^+^) (Equations (S38) and (S39)) were described in accordance with Sukhova et al. [70]. H^+^-ATPase was described based on the “two-state model” [77] (Equation (S18)); a regulation of its activity by blue light and ATP concentration in the cytoplasm [78] was included in the model using the Hill Equation (Equations (S19) and (S20)). We used a stationary description of this ATP concentration (Equation (S40)), which was based on the ATP synthesis dependent on the dark respiration (constant) and the CO_2_ assimilation rate (the FvCB model) and the ATP hydrolysis with the assumed velocity constant. 

K^+^ fluxes through inwardly and outwardly rectifying K^+^ channels were described based on the Goldman–Hodgkin–Katz Equation [76,79] (Equations (S21) and (S22)); the regulation of activities of these channels by the electrical potential across the plasma membrane of mesophyll cells was described based on the stationary solution of the Equation for the open probability for these channels [70] (Equations (S23) and (S24)).

H^+^ and K^+^ fluxes through the K^+^/H^+^-antiporter were described in accordance with our previous works [70,76] (Equation (S25)); this description was based on the simple Equation of the chemical kinetics. The K^+^/H^+^-antiporter was described as the electroneutral transporter because the transport of charges was compensated in this system.

The lateral fluxes of H^+^ and K^+^ were described based on Fick’s law in accordance with Sukhov et al. [80], (Equations (S31) and (S32)). The electrical potential across the plasma membrane was described as the stationary value in accordance with Sukhov et al. [80], (Equation (S26)); it was assumed that the electrical conductance between cells was zero. 

The developed model included numerous parameters that made it difficult for the direct experimental parameterization of a specific plant object. Considering this point, we mainly used standard parameters from the FvCB model [50] and from our previous model of ion transport and electrogenesis in plant cells [70] (Table S1 in File S1); other data from the literature were also used for the parameterization. As a result, this model could rather show the qualitive properties of forming spatial heterogeneity in the photosynthetic parameters in the leaf surface. In contrast, this model (with the current parameters) was not optimal for the quantitative predictions of the specific plant object. It should be additionally noted that using standard parameters, which provided good descriptions of investigated processes in earlier works, minimized the probability of qualitive errors in the results of the simulation. In contrast, the broad experimental search of parameters in specific species of plants could, potentially, induce these errors (strong experimental errors in the estimation of even one of numerous parameters can disrupt the results of a simulation).

The developed model was numerically analyzed using the forward Euler method. The special computer program (Microsoft Visual C++ 2019, Microsoft Corporation, Redmond, WA, USA) was developed for the numerical analysis of the model. Equation (1) was used for the calculation of the A_hv_ simulated by the developed model.

The action of excess light and drought on the spatial heterogeneity was analyzed in our work. The excess light action was provided by using the high values of the Photosynthetic Active Radiation (PAR) in Equation (S5). It was assumed that the drought action on plants was mainly related to the stomatal closure. At the model analysis, this action was imitated by using the decreased stomatal CO_2_ conductance (the decreased parameter g_S_^0^ in Equation (3), the quantity of open stomata per leaf area was not changed) or the decreased quantity of open stomata per leaf area (from one stomata per 3 × 3 elements square to one stomata per 5 × 5 elements square, the stomatal CO_2_ conductance was not changed). The average leaf CO_2_ conductance (g_S_) was decreased from 0.064 mol m^−2^s^−1^ to 0.023 mol m^−2^s^−1^ in both cases of the model analysis.

## 3. Results

### 3.1. Verification of the Developed Model on the Basis of Light Curves of Simulated and Experimental Photosynthetic CO_2_ Assimilation Rate

The first question of the current analysis was: could the developed model simulate the experimental light curve of the photosynthetic CO_2_ assimilation rate? We used the average photosynthetic CO_2_ assimilation rates in pea plant leaves under the actinic light with different intensities and these assimilation rates at different average leaf CO_2_ conductance for this verification. Experimental and simulated results were compared in a quality manner by using the standard parameters of the FvCB model [50], which were not adapted for pea plants. The details of the experimental procedures are described in Section 5 “Materials and Methods”.

**Figure 2:**
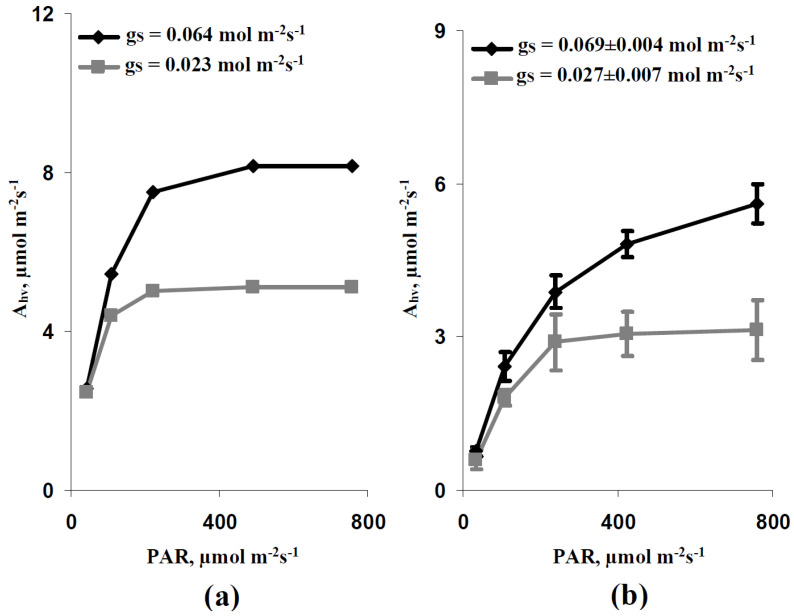
Simulated (**a**) and experimental (**b**) dependences of the average photosynthetic CO_2_ assimilation rate (A_hv_) on the intensity of the photosynthetic active radiation (PAR) at the varied average leaf CO_2_ conductance (g_S_). Simulated dependences were calculated at average g_S_ = 0.064 mol m^−2^s^−1^ (the basic g_S_) and g_S_ = 0.023 mol m^−2^s^−1^ (the decreased g_S_). Each stomata in the model was located in the center of square (3 × 3 elements); the average gS was calculated as the CO_2_ conductance in the element with stomata divided by 9. In order to obtain experimental dependences, all experimental records in this series were ranged and divided into two groups with the low (g_S_ < 0.04 mol m^−2^s^−1^, *n* = 5) and high (g_S_ > 0.04 mol m^−2^s^−1^, *n* = 9) CO_2_ conductance (see Section 5.1). A combination of Dual-PAM-300 and GFS-3000 was used in the experimental measurements of pea seedlings.

It is shown (Figure 2a) that the simulated dependence of average A_hv_ on the intensity of the actinic light at the basic g_S_ (0.064 mol m^−2^s^−1^) included two parts: the increase in the CO_2_ assimilation rate with increasing intensity of illumination (low and moderate light intensities) and the light saturation of this assimilation rate (high light intensities). This effect was also observed at the decreased average g_S_ (0.023 mol m^−2^s^−1^), which was imitated by using the decreased stomatal CO_2_ conductance; however, the maximal A_hv_ at g_S_ = 0.064 mol m^−2^s^−1^ was higher than one at g_S_ = 0.023 mol m^−2^s^−1^. Additionally, the minimal light intensity for the A_hv_ saturation at g_S_ = 0.064 mol m^−2^s^−1^ was higher than one at g_S_ = 0.023 mol m^−2^s^−1^.

Experimental plants were ranged in accordance with their g_S_ and were divided into two groups with high and low CO_2_ conductance (average g_S_ in leaves was 0.069 ± 0.004 and 0.027 ± 0.007 mol m^−2^s^−1^, respectively, see Section 5.1). It is shown (Figure 2b) that experimental A_hv_ dependences on light intensity were similar to simulated ones: (i) there were stages of increase in the photosynthetic CO_2_ assimilation rate and stage of A_hv_ light saturation, (ii) the maximal A_hv_ was increased with increasing g_S_, and (iii) the minimal light intensity for the A_hv_ saturation was increased with increasing stomatal CO_2_ conductance. It should be additionally noted that the values of the maximal A_hv_ differed in the experimental and the simulated results. This moderate quantitative difference could be caused by the used standard values of the model parameters, which were not adapted for pea seedlings (see Section 2).

**Figure 3:**
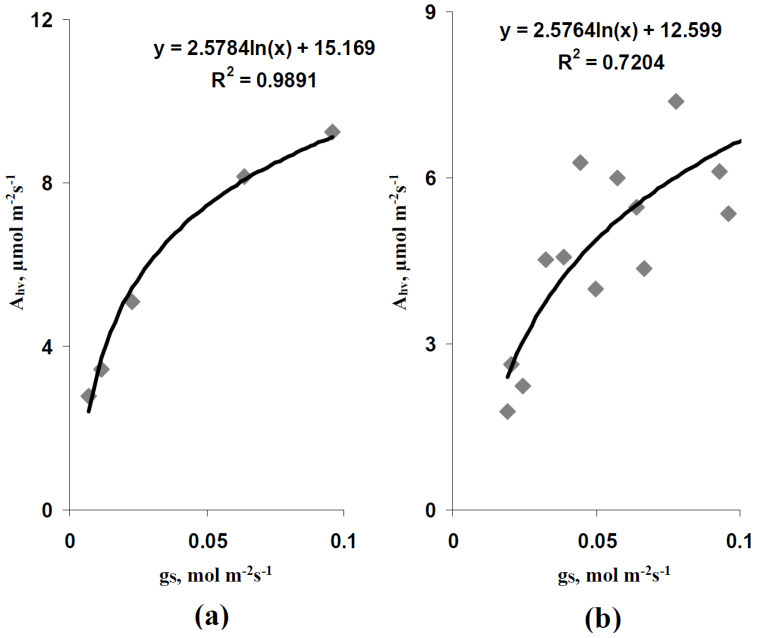
Simulated (**a**) and experimental (**b**) scatter plots between the average photosynthetic CO_2_ assimilation rate (A_hv_) and the average leaf CO_2_ conductance (g_S_) under high intensity of the photosynthetic active radiation (758 µmol m^−2^s^−1^). Simulated Ahv were calculated at the average g_S_ equaling 0.007, 0.012, 0.023, 0.064, and 0.096 mol m^−2^s^−1^. Each stomata in the model was located in the center of square (3 × 3 elements); the average g_S_ was calculated as the CO_2_ conductance in the element with stomata divided by 9. Pea seedlings were experimentally investigated; all g_S_ and A_hv_ (under the 758 µmol m^−2^s^−1^ PAR intensity) were used (*n* = 14). R^2^ is the determination coefficient.

Simulated (Figure 3a) and experimental (Figure 3b) dependences of A_hv_ on g_S_ at the high light intensity (758 µmol m^−2^s^−1^) were analyzed in the next stage of our work. It is shown that both dependences were qualitatively similar and could be described by logarithmic Equations with similar coefficients. Quantitative differences between dependences were probably caused by the absence of adaptation of parameters for pea plants.

Thus, these results showed that the developed model based on the two-dimensional system of photosynthetic cells could qualitatively describe important characteristics of A_hv,_ including the shape of the light dependence of the photosynthetic CO_2_ assimilation rate and changes in this shape and maximal A_hv_ during changes in the stomatal CO_2_ conductance. As a result, the developed model could be used for further analysis in our current work.

### 3.2. Analysis of Simulated and Experimental Spatial Heterogeneities in the Photosynthetic CO_2_ Assimilation Rate under Various Light Intensity and Stomatal CO_2_ Conductance

**Figure 4:**
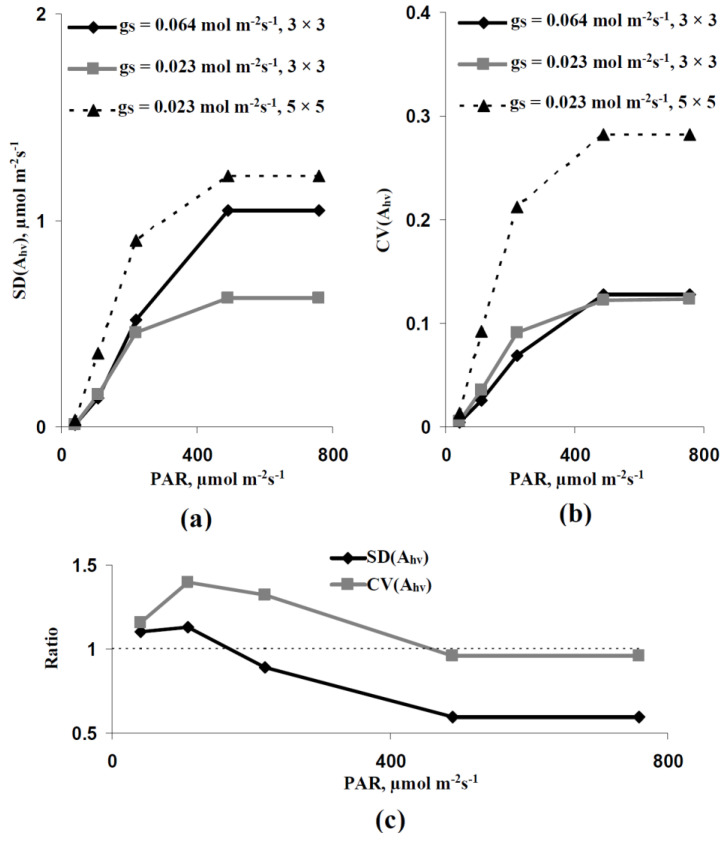
Dependences of parameters of the simulated spatial heterogeneity of the photosynthetic CO_2_ assimilation rate (A_hv_) on the intensity of the photosynthetic active radiation (PAR). (**a**) Dependence of the standard deviation of Ahv (SD(A_hv_)) on the PAR intensity. There were three variants of parameters. (i) The average g_S_ of the leaf was 0.064 mol m^−2^s^−1^, each stomata was located in the center of the 3 × 3 elements square. This variant was assumed as the control. (ii) The average g_S_ of the leaf was decreased to 0.023 mol m^−2^s^−1^. The CO_2_ conductance in individual stomata was decreased; each stomata was located in the center of the 3 × 3 elements square. (iii) The average g_S_ of the leaf was decreased to 0.023 mol m^−2^s^−1^. The CO_2_ conductance in individual stomata was not changed; each stomata was located in the center of the 5 × 5 elements square. (**b**) Dependence of the coefficient of variation of A_hv_ (CV(A_hv_)) on the PAR intensity. (**c**) Dependence of the ratio of the SD(Ahv) at g_S_ = 0.023 mol m^−2^s^−1^ (3 × 3 elements) to the SD(Ahv) at g_S_ = 0.064 mol m^−2^s^−1^ (3 × 3 elements) on the PAR intensity and the analogical dependence for CV(A_hv_).

The spatial heterogeneity of A_hv_ simulated by the developed model was analyzed in the next stage of investigation. First, the standard deviation of A_hv_ (SD(A_hv_)), which was calculated based on the values of A_hv_ in all elements of the two-dimensional model of the leaf, was analyzed. It is shown (Figure 4a) that SD(A_hv_) was increased with the increase in light intensity at all variants of the average leaf CO_2_ conductance. A decrease in the average g_S_ (from 0.064 to 0.023 mol m^−2^s^−1^) caused by the decrease in the stomatal CO_2_ conductance strongly decreased SD(A_hv_). In contrast, the similar decrease in the average g_S_ caused by the decrease in the quantity of stomata per area unit weakly influenced SD(A_hv_).

However, SD(A_hv_) should be strongly related to the absolute value of A_hv_; thus, all revealed changes could be related to changes in this value. We analyzed the coefficient of variation (CV(A_hv_)) to eliminate the influence of the absolute value of A_hv_ on the estimation of the spatial heterogeneity, because the variation coefficient was calculated as the standard deviation divided by the average value. It is shown (Figure 4b) that CV(A_hv_) was also strongly increased with increasing light intensity in all analyzed variants. The decrease in the average g_S_ caused by the decrease in the quantity of stomata per area unit strongly increased CV(A_hv_). The decrease in the average g_S_ caused by the decrease in the stomatal CO_2_ conductance weakly influenced CV(A_hv_); however, CV(A_hv_) in this variant was higher than CV(A_hv_) at the control average g_S_ (0.064 mol m^−2^s^−1^) under low and moderate light intensities.

We analyzed a ratio between SD(A_hv_) at the control average g_S_ and at the decreased average g_S_, which was caused by the decrease in the stomatal CO_2_ conductance (with no change in the quantity of stomata), and the analogical ratio between CV(A_hv_) to additionally estimate the last effect. It is shown (Figure 4c) that these ratios were increased under the low light intensity and the ratio of CV(A_hv_) was also increased under the moderate light intensity.

**Figure 5:**
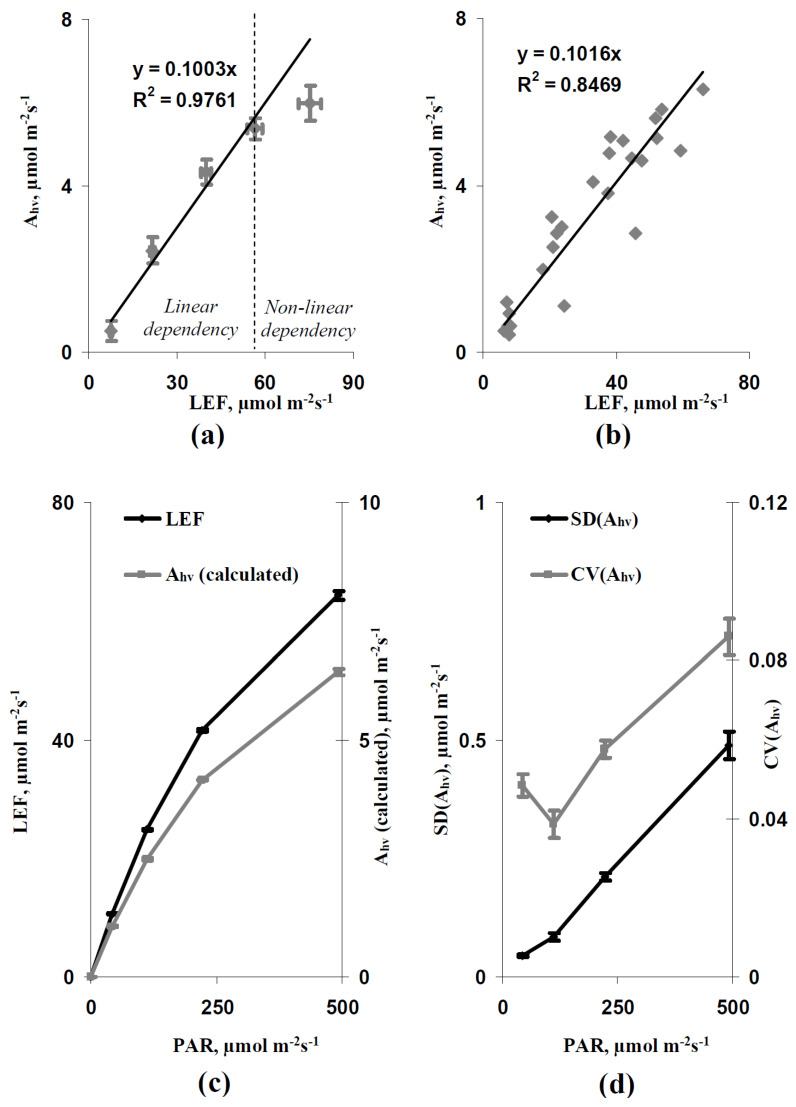
The dependence of average photosynthetic CO_2_ assimilation rate (A_hv_) on the average linear electron flow (LEF) at 34, 108, 239, 425, and 758 µmol m^−2^s^−1^ intensities of actinic light (*n* = 5–7) and the linear calibration Equation (**a**), the dependence of individual A_hv_ on individual LEF at 34, 108, 239, and 425 µmol m^−2^s^−1^ light intensities (*n* = 25) and the linear calibration Equation (**b**), dependences of LEF and A_hv_ (calculated) on the PAR intensity (*n* = 6) (**c**), and dependences of parameters of the spatial heterogeneity of Ahv (calculated) (SD(Ahv) and CV(A_hv_)) on the PAR intensity (*n* = 6) (**d**). R^2^ is the determination coefficient. Ahv (calculated) was calculated based on LEF and the calibration Equation. A combination of Dual-PAM-300 and GFS-3000 was used for development of the calibration Equation. IMAGING-PAM M-Series MINI Version was used for analysis of the spatial heterogeneity of A_hv_. Pea seedlings were used in all variants of experiments.

Thus, the results of the simulation show that the increase in light intensity and the decrease in leaf CO_2_ conductance could increase the spatial heterogeneity of the photosynthetic CO_2_ assimilation rate. After that, we experimentally analyzed this heterogeneity to check the revealed results. The direct experimental analysis of A_hv_ was not possible. However, the FvCB model [42,49,50,51] predicted that the linear relation between A_hv_ and LEF could be probable at the limitation of photosynthesis by the linear electron flow. Figure 5a shows that the average A_hv_ and LEF were strongly linearly related with increasing LEF (with increasing intensity of the actinic light) to about 60 µmol m^−2^s^−1^; this linear relation was disrupted at higher values of LEF (75 µmol m^−2^s^−1^ LEF at the 758 µmol m^−2^s^−1^ light intensity). Analysis of individual A_hv_ and LEF (excluding LEF at 758 µmol m^−2^s^−1^ light intensity) showed a similar linear relation at LEF equaling 6.5–66.2 µmol m^−2^s^−1^ (Figure 5b). Thus, linear regression A_hv_ = 0.1 LEF was used for the calculation of A_hv_ based on the measured LEF at LEF ≤ 66 µmol m^−2^s^−1^.

It is shown that the increase in light intensity increased the linear electron flow and calculated A_hv_ (Figure 5c). The experimental SD(A_hv_) and CV(A_hv_), which showed the spatial heterogeneity of the photosynthetic CO_2_ assimilation rate in leaves, were also increased with increasing light intensity (Figure 5d). This result was in good accordance with the results of the simulation and supported the induction of the photosynthetic spatial heterogeneity under excess light conditions.

**Figure 6:**
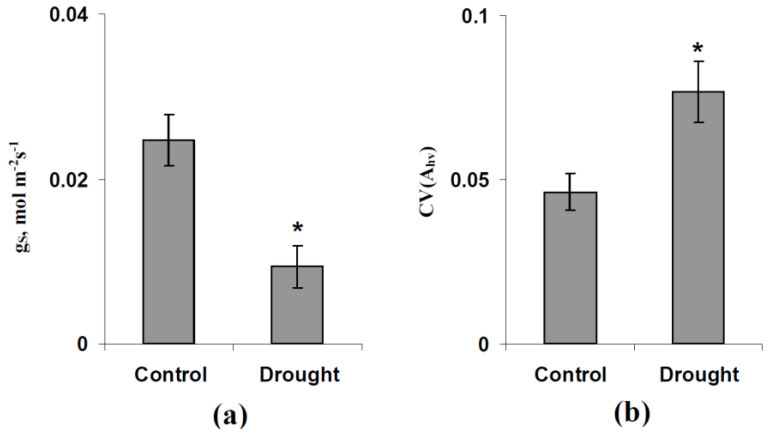
Influence of the short-term drought (1 day) on the leaf CO_2_ conductance (g_S_) (**a**) and the coefficient of variation of A_hv_ (CV(A_hv_)) showing the relative spatial heterogeneity of this parameter in the leaf (**b**) (*n* = 6). GFS-3000 was used for the gS measurement (averaged in the investigated area of the leaf) and IMAGING-PAM M-Series MINI Version was used for the analysis of the spatial heterogeneity of A_hv_ (based on the spatial heterogeneity of LEF and the calibration Equation). The moderate light intensity (249 µmol m^−2^s^−1^) was used in this experiment. Pea seedlings were irrigated in the control and were not irrigated under drought conditions. *, difference with the control was significant.

Finally, we experimentally checked the increase in CV(A_hv_) at the decreased average g_S_ that was predicted by the developed model. It is shown that the short-term drought (1 day) decreased the g_S_ in pea leaves (Figure 6a), which was probably related to the stomata closing. CV(A_hv_), calculated based on the variation coefficient of LEF, was significantly increased during the short-term drought (Figure 6b). This result experimentally supported the increase in photosynthetic spatial heterogeneity due to the stomata closing.

## 4. Discussion

Photosynthesis is a complex process [1,2] that can be strongly affected by numerous abiotic stressors [3,4,15,16]. The simulation of photosynthetic processes is an effective prediction tool of photosynthetic changes under the action of stressors [31]. There are photosynthetic models focusing on descriptions of the primary light absorption [32,33,34], photosynthetic light reactions [5,35,36,37,38,39,40], photosynthetic dark reactions, and CO_2_ fluxes [41,42,43,44], etc. However, mathematical models of photosynthetic processes in the scale of the leaf surface, which can be used for revealing the spatial heterogeneity of the distribution of photosynthetic parameters on this surface, are weakly developed. Our current work—devoted to the solution of this problem—shows two important results.

First, the developed two-dimensional model of C_3_ photosynthesis in the leaf, which is based on the FvCB model [42,49,50,51], descriptions of stomatal and transmembrane fluxes of CO_2_ and lateral fluxes of CO_2_ and HCO_3_^−^ [73,74,75], and the simplified model of the H^+^ and K^+^ transport [70,76,77,80] can qualitatively simulate the experimental results, including the shape of dependence of the average A_hv_ in the leaf on the light intensity and the influence of the average g_S_ on the photosynthetic CO_2_ assimilation rate (see Figure 2 and Figure 3). It is important that this accordance between the experimental and the simulated results does not require additional adaptation of parameters of the photosynthetic description in the developed model because standard parameters of the FvCB model [50] are used (Table S1 in File S1). This result verifies the efficiency of the developed model for the simulation of the average A_hv_. Furthermore, considering that this model can also describe the spatial heterogeneity of the A_hv_ distribution on the leaf surface, it is a potential tool for the investigation of the influence of stressors on this heterogeneity.

Second, the developed model predicts the increase in the A_hv_ spatial heterogeneity on the leaf surface with increasing light intensity (Figure 4). This effect is related to the stomatal CO_2_ conductance and the quantity of open stomata supporting the CO_2_ flux into the leaf, because the decrease in this conductance or the quantity of open stomata per leaf area increases the simulated photosynthetic spatial heterogeneity (especially at the weak and moderate light intensities). The results of analysis of the developed model are in good accordance with works showing the relations of the spatial heterogeneity and the dynamics of the stomata opening to the distribution of photosynthetic parameters in leaves [81,82,83]. Additionally, there are works [83,84,85,86] showing an increase in the spatial heterogeneity of photosynthetic parameters under drought conditions. The participation of the stomata closing due to this effect is a discussion question [85,86]; however, considering the influence of drought on stomata [87,88], this participation cannot be excluded.

Our experimental results support the prediction of the developed model: an increase in light intensity increases the variation coefficient of the photosynthetic CO_2_ assimilation rate in pea leaves (Figure 5d) and a decrease in leaf CO_2_ conductance, induced by the short-term drought, also increases this coefficient (Figure 6). These results, which are in good accordance with the noted experimental works by other authors showing the positive drought influence on the photosynthetic spatial heterogeneity in leaves [83,84,85,86] additionally verify the developed model.

A potential mechanism of the revealed light-induced increase in the A_hv_ spatial heterogeneity can be related to the heterogeneity of the stromal CO_2_ concentration in the different cells. In accordance with the FvCB model [42,49,50,51], this concentration can strongly influence A_hv_ in cells. On the other hand, CO_2_ is propagated from stomata through lateral diffusion [89,90] and is consumed by photosynthetic processes, which can be dependent on the light intensity. It means that an increase in this intensity and the stimulation of photosynthesis should increase the variability of the CO_2_ concentration in different cells; i.e., the light intensity should influence the spatial heterogeneity of the stromal CO_2_ concentration. The additional model analysis of the variation coefficient of this concentration shows that this coefficient is strongly increased by changes in the light intensity from 42 µmol m^−2^s^−1^ to 221 µmol m^−2^s^−1^ (from 0.013 to 0.100, respectively); thus, this mechanism can participate in an increase in the A_hv_ spatial heterogeneity under the excess light.

A decrease in the quantity of open stomata per leaf area should stimulate this effect by increasing the distance of the CO_2_ diffusion. This supposition is supported by an increase in the variation coefficient of the simulated stromal CO_2_ concentration from 0.100 to 0.180 by decreasing this quantity from one stomata per 9 cells to one stomata per 25 cells under the 221 µmol m^−2^s^−1^ light intensity. In contrast, a decrease in the stomatal CO_2_ conductance (without changes in the open stomata quantity) weakly influences this coefficient (data not shown). The last result shows that there are additional induction mechanisms of the A_hv_ spatial heterogeneity in the leaf. It cannot be excluded that these additional mechanisms also participate in influencing the light intensity on the A_hv_ heterogeneity. 

The revealed stimulation of the A_hv_ spatial heterogeneity under excess light conditions and/or under the decreased leaf CO_2_ conductance (imitation of the drought) can potentially modify the non-photochemical quenching of the chlorophyll fluorescence, including photodamage, state-transition in the light-harvesting complex, and energy-dependent quenching [3,4,18,19], because low A_hv_ in some parts of a leaf can strongly limit photosynthetic light reactions and can contribute to the induction of these processes. It means that this spatial heterogeneity can potentially modify the plant tolerance to the actions of the excess light. Particularly, cells with low CO_2_ concentration in the stroma and weak activity of the photosynthetic CO_2_ assimilation should have a low threshold for both photodamage and induction of protective changes in the photosynthetic machinery. It can be expected that these cells can influence damage and tolerance of whole leaves under the action of stressors (e.g., through the production and propagation of reactive oxygen species [71]); however, this supposition requires further development of the model (e.g., a description of the damage of photosynthetic machinery in the model can be included in the model) and the model-based investigations. 

Additionally, the increased A_hv_ spatial heterogeneity and related changes in photosynthetic light reactions can be used for the development of methods of remote sensing plant stress changes under excess light or drought conditions. Particularly, it can be expected that these stressors should increase the heterogeneity of the spatial distribution of PRI because this reflectance index is strongly related to photosynthetic parameters [61,62,64,66,67]. Potentially, this effect can be used for the development of methods of remote sensing the actions of excess light and drought on plants (based on the measurements of the spatial heterogeneity of PRI); however, this possible stimulation of PRI under the action of stressors requires future model-based and experimental investigations.

**Figure 7:**
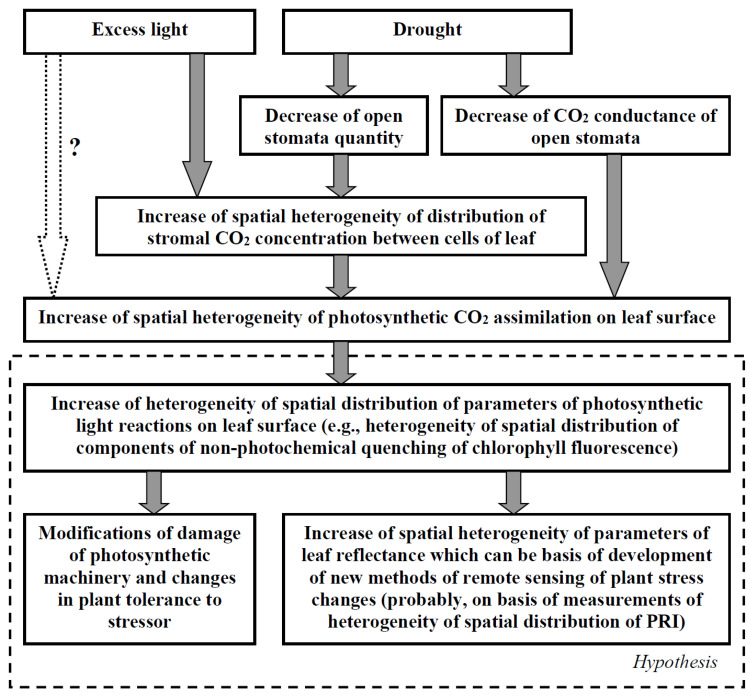
A scheme of potential ways the excess light and drought influencing the heterogeneity of the spatial distribution of photosynthetic parameters and the hypothetical importance of this heterogeneity for the plant tolerance and remote sensing of plant stress changes. The scheme is based on analysis of the developed model and experimental results (see Section 4 for details).

Figure 7 summarizes the results of our work and their potential importance for understanding the ways of plant damage and tolerance under the action of stressors and the development of methods for plant remote sensing. It should be additionally noted that the developed model can be used for future analysis of the influence of the stochastic spatial heterogeneity of its parameters on photosynthetic processes; e.g., the influence of the stochastic heterogeneity of the activity of H^+^-ATPases in the plasma membrane [31], which is related to the CO_2_ flux into mesophyll cells [71], or the influence of the stochastic heterogeneity of the CO_2_ conductance of individual stomata can be investigated. It is known that the stochastic spatial heterogeneity of biological objects (including plants) can influence their systemic parameters (e.g., through “diversity-induced resonance” or similar effects, [31,68,69]); thus, the analysis of this problem based on the developed model can be an important task.

Other interesting perspectives of the model development can be: description of stomata regulation mechanisms by light intensity and drought (and potential interactions between these mechanisms), description of the light damage to photosynthetic machinery (and relation of this damage with stomata opening, the plasma membrane and chloroplast envelope CO_2_ conductance, and activity of the CO_2_ carboxylation), and description of the influence of photosynthetic processes to leaf reflectance (this description can be important for the development of methods of remote sensing). Finally, the parameterization of the model for specific plant species (e.g., plant species that are widely used in agriculture) can be an additional important task for the future development of the model.

## 5. Materials and Methods

### 5.1. Experimental Procedure of Verification of Two-Dimensional Model of the C_3_ Photosynthesis in Plant Leaves

We did not parameterize the two-dimensional model of C_3_ photosynthesis in leaves for the specific plant, because using the standard parameters from earlier models, which were included in the current model, simplified parameterization and minimized potential errors in parameter values that were probable at the broad experimental search and could disrupt the model analysis.

Therefore, we could not expect a quantitative accordance between the simulated and the experimental photosynthetic parameters at verification. As a result, we analyzed the qualitive accordance between the results of the simulation and the results of the experimental investigation of the pea plant. Pea plants were selected based on our numerous early works, which investigated photosynthesis and its regulation in this plant object (e.g., [5,66,67,91]).

Thus, 2–3-week-old pea seedlings (*Pisum sativum* L., cultivar “Albumen”) were used for verification of the two-dimensional model of C3 photosynthesis in plant leaves. The plants were cultivated in a sand substrate in a Binder KBW 240, with irrigation by the 50% Hoagland–Arnon medium (about 50 mL) performed every two days. Luminescent lamps FSL YZ18RR (Foshan Electrical And Lighting Co., Ltd., Foshan, China) were used for illumination (about 100 µmol m^−2^s^−1^). The weak water deficit (the short-term drought) was induced by an absence of irrigation of the experimental seedlings for 1 day.

A combination of a PAM-fluorometer Dual-PAM-100 and an infrared gas analyzer GFS-3000 (Heinz Walz GmbH, Effeltrich, Germany) was used for the investigation of the average photosynthetic parameters in the second mature leaves of the pea plant. A_hv_ was measured as the difference between the CO_2_ assimilation rate after 10 min under the actinic blue light (Dual-PAM-100 was used as the source of this light) and this assimilation rate under dark conditions. The current CO_2_ assimilation rate was measured by the gas analyzer GFS-3000. The leaf CO_2_ conductance was calculated based on the leaf water conductance, which was measured by GFS-3000, in accordance with Cabrera et al. [92]. The GFS-3000 was also used for supporting the 360 ppm concentration of CO_2_ and the 70% relative air humidity in the measuring cuvette.

A photosynthetic linear electron flow (LEF) was calculated based on the effective quantum yield of the photosystem II (Φ_PSII_), the intensity of the actinic light (PAR), the fraction of absorbed light distributed to the photosystem II (dII = 0.42), and the fraction of PAR absorbed by the leaves (*p* = 0.88) in accordance with Equation (7) [91]:

(7) $$\mathrm{LEF}=p\cdot \mathrm{dII}\cdot \Phi_{\mathrm{PSII}}\cdot \mathrm{PAR}$$

Φ_PSII_ was estimated after 10 min under the actinic light. This parameter was automatically calculated by the Dual-PAM-100 software based on the current levels of fluorescence (F) and the maximal fluorescence level after the preliminary illumination ($F_{m}^{\prime }$), which were measured before initiation and before termination of the saturation pulse (300 ms, red light, 10,000 µmol m^−2^s^−1^), respectively, in accordance with the standard procedure of measurement by the PAM fluorometer. Equation (8) was used for the Φ_PSII_ calculation [93]:

(8) $$\Phi_{\mathrm{PSII}}=\frac{F_{m}^{\prime }-F}{F_{m}^{\prime }}$$

The blue light from the standard source of Dual-PAM-100 was used as the actinic light; its intensity was varied.

There were two variants of experiments combining the Dual-PAM-100 and the GFS-3000. First, we preliminary experimentally estimated the basic g_S_ that was used for the calculation of the stomatal CO_2_ conductance in the model (g_S0_ = g_S_·9 because one stomata per nine elements was used as the control variant in the model, Table S1 in File S1). Experiments were performed for 1 day; light curves were not analyzed. It was shown that g_S_ = 0.064 ± 0.04 mol m^−2^s^−1^ (*n* = 6). As a result, g_S_ = 0.064 mol m^−2^s^−1^ (and g_S0_ = 0.576 mol m^−2^s^−1^) was used as the basic leaf CO_2_ conductance. In the model, the decreased g_S_ was provided by the decreased g_S_^0^ or the decreased quantity of stomata per leaf area (from one stomata per 3 × 3 elements square to one stomata per 5 × 5 elements square, see Section 2); both decreased g_S_ should be the same when compared. Thus, the decreased g_S_ was calculated as the multiplication between the basic g_S_ and 9/25 (the decreased g_S0_ was similarly calculated, Table S1 in File S1).

Second, we analyzed the experimental light curves, which were investigated for the long-time experimental series (about 2 weeks). In this case, the experimental g_S_ was more varied than the g_S_ in the first case (g_S_ = 0.058 ± 0.11 mol m^−2^s^−1^, *n* = 14). This variability was used for the additional verification of the model; all experimental records in this series were ranged and divided into two groups with the low (g_S_ < threshold value) and high (g_S_ > threshold value) CO_2_ conductance. We found that using the 0.04 mol m^−2^s^−1^ threshold value provided an average g_S_ which was similar to the leaf CO_2_ conductance in the model: 0.069 ± 0.004 mol m^−2^s^−1^ (*n* = 9) and 0.027 ± 0.007 mol m^−2^s^−1^ (*n* = 5). After that, we separately statistically analyzed the light dependences in these two groups (with the low and high CO_2_ conductance) to verify the developed model. 

A system of PAM imaging IMAGING-PAM M-Series MINI Version (Heinz Walz GmbH, Effeltrich, Germany) was used for the measurements of the spatial distribution of photosynthetic parameters. The blue light from the standard source of this system was used as the actinic light; its intensity was varied. Φ_PSII_ was estimated at the saturation pulse (in accordance with Equation (8)) after 10 min under the actinic light.

The analysis of the spatial distributions of LEF was based on the analysis of grayscale images of the spatial distribution of the quantum yield of photosystem II, which were created by software of the IMAGING-PAM M-Series MINI Version. These grayscale images were analyzed using ImageJ 1.46r. The analysis showed the average value and the standard deviation of Φ_PSII_ in the standard round ROI in the center of the leaf. The coefficient of variation was calculated as the ratio of the standard deviation of the average value. The parameters of LEF (the average value, standard deviation, and coefficient of variation) were calculated based on Equation (7) as the simple proportion. These parameters were used for the estimation of the parameters of A_hv_ (the average value, standard deviation, and coefficient of variation) based on the calibration curve (see Section 3.2).

### 5.2. Statistics

Means and standard errors were used in the statistical analysis and Student’s *t*-test was used for the estimation of significance. The spatial heterogeneity was estimated based on the standard deviation of A_hv_ (SD(A_hv_)) and the coefficient of variation of this photosynthetic parameter (CV(A_hv_)). Numbers of repetitions were shown in figures.

## 6. Conclusions

The work was devoted to the development of a two-dimensional model of C_3_ photosynthesis in the plant leaf and further analysis of the induction of the spatial heterogeneity of the CO_2_ assimilation rate under the excess light and a decrease in the leaf CO_2_ conductance; this g_S_ decrease imitated the action of a short-term drought. First, it was shown that the developed two-dimensional model of C_3_ photosynthesis in the leaf (based on the FvCB model, the descriptions of the fluxes of CO_2_ and HCO_3_^−^, and the simplified model of the H^+^ and K^+^ transport) qualitatively simulated the experimental results. Second, the analysis of the developed model showed that the increase in the light intensity and the decrease in the average leaf CO_2_ conductance should increase the spatial heterogeneity of the photosynthetic CO_2_ assimilation rate on the leaf surface. Experimental investigations supported these theoretical results. Thus, the developed model can be used as a tool for theoretical investigations of the influence of environmental factors on the spatial heterogeneity of the distribution of photosynthetic parameters in the leaf. Finally, there are some potential ways to further develop the model, including its parameterization for specific plant species, additional description of stomata regulation by light and drought, description of light damage to photosynthetic machinery, description of relations between photosynthesis and leaf reflectance, analysis of influence of stochastic heterogeneity in photosynthetic and stomata parameters, and others.

## Data Availability

The data presented in this study are available upon request from the corresponding author.

## Supplementary Materials

The following supporting information can be downloaded at: https://www.mdpi.com/article/10.3390/plants11233285/s1, File S1 “Equations and parameters of the two-dimensional photosynthetic model”, Refs [50,51,70,72,73,74,75,76,77,78,79,80,94,95,96,97,98] have mentioned in Supplementary Materials.
