# Supplementary material for: Development of Two-Dimensional Model of Photosynthesis in Plant Leaves and Analysis of Induction of Spatial Heterogeneity of CO2 Assimilation Rate under Action of Excess Light and Drought
Source: Plants (Basel). 2022 Nov 29;11(23):3285. doi: 10.3390/plants11233285 (PMC9739240; doi:10.3390/plants11233285)
Supplement: Supplementary file 1 [file plants-11-03285-s001.zip › plants-1991941-file S1.pdf]

Development of Two-Dimensional Model of Photosynthesis in Plant Leaves and Analysis of Induction of Spatial Heterogeneity in CO_2 Assimilation Rate under Action of Excess Light and Water Shortage

Ekaterina Sukhova, Daria Ratnitsyna, Ekaterina Gromova, Vladimir Sukhov *

Department of Biophysics, N.I. Lobachevsky State University of Nizhny Novgorod, Nizhny Novgorod, 603950, Russia
* Corresponding: vssuh@mail.ru

Supplementary Materials

Equations and Parameters of the Two-Dimensional Photosynthetic Model

1. Description of photosynthetic CO_2 assimilation and photorespiration

The simplified photosynthetic model of Farquhar, von Caemmerer and Berry (FvCB-model) [1-2] which included only the Rubisco-limited CO_2 assimilation rate and electron transport-limited CO_2 assimilation rate was used in the work. The assimilation (A_{hv}) was described by Equation (S1):

$$A_{hv} = \min(W_c, W_j)\frac{[CO_2]_{str} - \Gamma^*}{[CO_2]_{str}} \quad (S1),$$

where W_c (Equation (S2)) and W_j (Equation (S3)) were carboxylation rates at the Rubisco-limited CO_2 assimilation and electron transport-limited CO_2 assimilation conditions, respectively, $[CO_2]_{str}$ was concentration of CO_2 in the stroma of chloroplasts, Γ^* was the photosynthetic CO_2 compensation point in the absence of mitochondrial respiration.

$$W_c = \frac{V_{max}[CO_2]_{str}}{[CO_2]_{str} + K_C\left(1+\frac{[O_2]}{K_O}\right)} \quad (S2),$$

$$W_j = \frac{J}{4+8\frac{\Gamma^*}{[CO_2]_{str}}} \quad (S3),$$

where V_{max} was the maximum rate of carboxylation of Rubisco, $[O_2]$ was concentration of O_2 in the stroma of chloroplasts, K_C and K_O were the Michaelis constants for carboxylation and oxygenation, respectively, J was the potential of whole chain electron transport (Equation (S4)):

$$J = \frac{I + J_{max}}{2\theta} - \frac{\sqrt{(I + J_{max})^2 - 4\theta I J_{max}}}{2\theta} \quad (S4),$$

where I (Equation (S5)) was the useful light absorbed by the photosystem II, J_{max} was the maximum electron transport rate, and θ was an empirical curvature factor [1].

$$I = \frac{abs(1-f)}{2}PAR \quad (S5),$$

where PAR was the photosynthetic active radiation, abs was the light absorptance (abs), and f was the factor of correction for spectral quality of the light.

The photorespiration rate (V_{phr}) was described by Equation (S6):

$$V_{phr} = \frac{A_{h\nu}\Gamma^*}{[CO_2]_{str}} \quad (S6),$$

In accordance with von Caemmerer et al. [1], it was assumed that rate of the dark respiration (R_d) was constant.

2. Description of stomata and transmembrane CO_2 fluxes

Equations (S7), (S8), and (S9) based on the Fick's law [3] were used for description of CO_2 transport through the stomata (j_S), plasma membrane (j_{PM}), and chloroplast envelopes (j_{Chl}), respectively:

$$j_S = g_S{}^0([CO_2]_{out} - [CO_2]_{ap}) \quad (S7),$$

$$j_{PM} = g_{PM}([CO_2]_{ap} - [CO_2]_{cyt}) \quad (S8),$$

$$j_{Chl} = g_{Chl}([CO_2]_{cyt} - [CO_2]_{str}) \quad (S9),$$

where $[CO_2]_{out}$, $[CO_2]_{ap}$, and $[CO_2]_{cyt}$ were concentrations of CO_2 in the air, apoplast and cytoplasm, respectively, $g_S{}^0$, g_{PM}, and g_{Chl} were CO_2 conductance for the stomata, plasma membrane, and chloroplast envelopes, respectively.

It was assumed that g_S was constant in model elements with both mesophyll cell and stomata or zero in model elements without stomata. It was also assumed that the CO_2 conductance per area unit was equal for the plasma membrane and chloroplast envelopes [4-5]; thus, g_{PM} and g_{Chl} were derived from the CO_2 conductance through mesophyll (g_m):

$$g_{Chl} = \frac{g_m\left(1 + \frac{S_{PM}}{S_{Chl}}\right)}{\frac{S_{PM}}{S_{Chl}}} \quad (S10),$$

$$g_{pm} = g_m\left(1 + \frac{S_{PM}}{S_{Chl}}\right) \quad (S11),$$

where $\frac{S_{PM}}{S_{Chl}}$ was ratio of the total area of the plasma membranes to the total area of the envelopes of chloroplasts.

3. Description of lateral fluxes of CO_2 and HCO_3^-

Equations (S12) and (S13) based on our previous work [6] were used for description of CO_2 ($j_{CO2}{}^{n,k/l.m}$) and HCO_3^- ($j_{HCO3}{}^{n,k/l.m}$) volume fluxes, respectively, between apoplasts of two neighboring cells (their parameters were marked by indices n, k and l, m):

$$j_{CO2}{}^{n,k/l,m} = \frac{D_{CO2}}{a^2(1 + \frac{V_{ap}}{V_{cell}})^{1/3}}([CO_2]_{ap}{}^{l,m} - [CO_2]_{ap}{}^{n,k}) \quad (S12),$$

$$j_{HCO3}{}^{n,k/l,m} = \frac{D_{HCO3}}{a^2(1 + \frac{V_{ap}}{V_{cell}})^{1/3}}([HCO_3{}^-]_{ap}{}^{l,m} - [[HCO_3{}^-]]_{ap}{}^{n,k}) \quad (S13),$$

where $[HCO_3^-]_{ap}$ was concentration of HCO_3^- in the apoplast, D_{CO2} and D_{HCO3} were coefficients of diffusion of CO_2 and HCO_3^- in water, and a was linear size of cell in the model (it was assumed that these cells were

cubes), $\frac{V_{ap}}{V_{cell}}$ was ratio of the apoplastic volume to the total cell volume. In border elements, CO_2 and HCO_3^- fluxes directed outside of the simulated leaf were assumed equaling to zero.

4. Description of changes in CO_2 and HCO_3^- concentrations

Equations (S14), (S15), and (S16) were used for calculation of summary changes in CO_2 and HCO_3^- concentrations in the apoplast (C_{ap}), cytoplasm (C_{cyt}), and chloroplast stroma (C_{str}), respectively:

$$\frac{dC_{ap}{}^{n,k}}{dt} = 18\cdot 10^{-5}\frac{S_{leaf}}{V_{ap}}(j_S{}^{n,k} - j_{PM}{}^{n,k}) + j_{CO2}{}^{n,k/n-1,k} + j_{CO2}{}^{n,k/n+1,k} + j_{CO2}{}^{n,k/n,k-1} + + j_{CO2}{}^{n,k/n,k+1} + j_{HCO3}{}^{n,k/n-1,k} + j_{HCO3}{}^{n,k/n+1,k} + j_{HCO3}{}^{n,k/n,k-1} + j_{HCO3}{}^{n,k/n,k+1} \quad \text{(S14),}$$

$$\frac{dC_{cyt}{}^{n,k}}{dt} = 18\cdot 10^{-5}\frac{S_{leaf}}{V_{cyt}}(j_{PM}{}^{n,k} - j_{Chl}{}^{n,k} + R_d + V_{phr} \quad \text{(S15),}$$

$$\frac{dC_{str}{}^{n,k}}{dt} = 18\cdot 10^{-5}\frac{S_{leaf}}{V_{str}}(j_{Chl}{}^{n,k} - \min(W_c, W_j)) \quad \text{(S16),}$$

where $\frac{S_{leaf}}{V_{ap}}$, $\frac{S_{leaf}}{V_{cyt}}$, and $\frac{S_{leaf}}{V_{str}}$ were ratios of leaf areas to volumes of the apoplast, cytoplasm, and stroma, respectively, $18\cdot 10^{-5}$ dm^3 mol^{-1} was volume of 1 mol of H_2O.

In accordance with our previous work [7], we used portion of CO_2 in the summary concentration of CO_2 and HCO_3^- (P_{CO2}) for calculation of separate concentrations of CO_2 and HCO_3^-. P_{CO2} was calculated on basis of Equation (S17):

$$P_{CO_2} = \frac{1}{1 + 10^{pH-pK}} \quad \text{(S17),}$$

where pK was the negative logarithm of the equilibrium constant in the reaction of transition between CO_2 and HCO_3^-. CO_2 concentrations were calculated as multiplication of P_{CO2} and the summary concentration of CO_2 and HCO_3^-; HCO_3^- concentrations were calculated as multiplication of (1-P_{CO2}) and the summary concentration of CO_2 and HCO_3^-.

5. Description of transmembrane H^+ and K^+ fluxes and membrane potential

Description of transmembrane H^+ and K^+ fluxes was based on our previous model [8-9]; however, this model was simplified. Only, H^+-ATPase, inwardly and outwardly rectifying K^+ channels, and K^+/H^+-antiporter were described.

Proton flux through H^+-ATPase (j_P) was described by Equation (S18) (in accordance with the "two-state model" [8, 10]):

$$j_P = A_{ATP}A_{BL}\frac{k_{+1}k_{+2} - k_{-1}k_{-2}}{k_{+1}+k_{+2} + k_{-1} + k_{-2}} \quad \text{(S18),}$$

where $k_{+1} = k_1[H^+]_{ap}$, $k_{-1} = k_1\exp\left(\frac{G_{ATP}}{RT}\right)$, $k_{+2} = \frac{k_2u}{1-\exp(-u)}$, and $k_{-2} = \frac{k_2u[H^+]_{cyt}\exp(-u)}{1-\exp(-u)}$ were velocity constants for transitions between states of the H^+-ATPase, A_{ATP} (Equation (S19)) and A_{BL} (Equation (S20)) were coefficients describing activation of H^+-ATPase by the cytoplasmic ATP concentration ([ATP]) and intensity of blue light (BL), respectively, $u = \frac{E_mF}{RT}$ was the normalized membrane potential, $[H^+]_{ap}$ and $[H^+]_{cyt}$ were proton concentrations in the apoplast and cytoplasm, respectively, G_{ATP} was the energy of ATP hydrolysis, k_1 and k_2 were are velocity constants of transitions between states of the H^+-ATPase at u=0 and $[H^+]_{ap} = [H^+]_{out}$ = 1 M, E_m was membrane potential across the plasma membrane, F, R, and T were standard thermodynamic values.

$$A_{ATP} = \frac{K_{cyt}[ATP]}{K_{ATP} + K_{cyt}[ATP]} \quad \text{(S19),}$$

$$A_{BL} = \frac{BL}{BL + K_{BL}} + A_{BL0}\frac{K_{BL}}{K_{BL} + BL} \quad \text{(S20),}$$

where K_{ATP} was the constant of the 50% activation of H^+-ATPase by [ATP], K_{cyt} was the proportional coefficient between concentrations of ATP in the leaf and cytoplasm, A_{BL0} was activity of H^+-ATPase without the blue light, K_{BL} was the constant of the 50% activation of H^+-ATPase by BL.

K^+ fluxes through inwardly (J_{IRKC}) and outwardly (J_{ORKC}) rectifying K^+ channels were described on basis of the Goldman–Hodgkin–Katz equation [8-9, 11]:

$$J_{IRKC} = \frac{P_{IRKC}P_{max}{}^{IRKC}u\left([K^+]_{cyt} - [K^+]_{ap}\exp(-u)\right)}{1 - \exp(-u)} \quad \text{(S21),}$$

$$J_{ORKC} = \frac{P_{ORKC}P_{max}{}^{ORKC}u\left([K^+]_{cyt} - [K^+]_{ap}\exp(-u)\right)}{1 - \exp(-u)} \quad \text{(S22),}$$

where P_{IRKC} (Equation (S23)) and P_{ORKC} (Equation (S24)) were probabilities of open states of inwardly and outwardly rectifying K^+ channels, respectively, where $P_{max}{}^{IRKC}$ and $P_{max}{}^{ORKC}$ were maximum permeabilities of inwardly and outwardly rectifying K^+ channels, respectively, $[K^+]_{cyt}$ and $[K^+]_{ap}$ were concentrations of K^+ in the cytoplasm and apoplast.

$$P_{IRKC} = \frac{1}{1 + \exp\left(c_{IRKC}(u - u_{IRKC})\right)} \quad \text{(S23),}$$

$$P_{IRKC} = \frac{1}{1 + \exp\left(c_{IRKC}(u_{IRKC} - u)\right)} \quad \text{(S24),}$$

where c_{IRKC} and c_{ORKC} were constants which represented a portion of the membrane potential acting on the gating mechanisms and their charge in inwardly and outwardly rectifying K^+ channels, respectively, u_{IRKC} and u_{ORKC} were the normalized potential barriers for the transition of the channel from the closed state to the open one in inwardly and outwardly rectifying K^+ channels, respectively.

H^+ and K^+ fluxes through K^+/H^+-antiporter (J_{Ant}) were described in accordance with our previous works [8-9]:

$$J_{Ant} = k_{Ant}\left([K^+]_{cyt}[H^+]_{ap} - [K^+]_{ap}[H^+]_{cyt}\right) \quad \text{(S25),}$$

where k_{Ant} was parameter which was proportional to rate of transports of ions through the antiporter.

E_m was described as stationary value in accordance with our previous works [6, 9]:

$$E_m = \frac{g_K E_K + g_P E_P}{g_K + g_P} \quad \text{(S26),}$$

where E_K and E_P were reverse potentials for K^+ channels (Equation (S27)) and H^+-ATPase (Equation (S28)), respectively, g_K and g_P were electrical conductance for K^+ channels (Equation (S29)) and H^+-ATPase (Equation (S30)), respectively:

$$E_K = \frac{RT}{F}\ln\left(\frac{[K^+]_{ap}}{[K^+]_{cyt}}\right) \quad \text{(S27),}$$

$$E_P = \frac{RT}{F}\ln\left(\frac{[H^+]_{ap}}{[H^+]_{cyt}}\right) + \frac{G_{ATP}}{F} \quad \text{(S28),}$$

$$g_K = \frac{F\left(J_{kg} + J_{kd}\right)}{E_m - E_K} \quad \text{(S29)},$$

$$g_P = \frac{FJ_{\mathrm{P}}}{E_m - E_P} \quad \text{(S30)},$$

6. Description of lateral H^+ and K^+ fluxes

Equations (S31) and (S32) based on our previous work [6] were used for description of K^+ ($j_K^{n,k/l.m}$) and H^+ ($j_H^{n,k/l.m}$) volume fluxes, respectively, between apoplasts of two neighboring cells (their parameters were marked by indices n, k and l, m):

$$\mathrm{j_K}^{\mathrm{n,k/l,m}} = \frac{\mathrm{D_K}}{\mathrm{a}^2(1 + \frac{\mathrm{V_{ap}}}{\mathrm{V_{cell}}})^{1/3}} ([K^+]_{\mathrm{ap}}{}^{\mathrm{l,m}} - [K^+]_{\mathrm{ap}}{}^{\mathrm{n,k}}) \quad \text{(S31)},$$

$$\mathrm{j_H}^{\mathrm{n,k/l,m}} = \frac{\mathrm{D_H}}{\mathrm{a}^2(1 + \frac{\mathrm{V_{ap}}}{\mathrm{V_{cell}}})^{1/3}} ([H^+]_{\mathrm{ap}}{}^{\mathrm{l,m}} - [[H^+]]_{\mathrm{ap}}{}^{\mathrm{n,k}}) \quad \text{(S32)},$$

where D_K and D_H were coefficients of diffusion of K^+ and H^+ in water. In border elements, ion fluxes directed outside of the simulated leaf were assumed equaling to zero.

7. Description of changes in K+ and H+ concentrations

Equations (S33), (S34), (S35), and (S36) were used for calculation of summary changes in total K concentrations in the apoplast ($[K]_{ap}$) and concentration of K^+ in the cytoplasm ($[K^+]_{cyt}$) and changes in total H concentrations in the apoplast ($[H]_{ap}$) and cytoplasm ($[H]_{cyt}$), respectively.

$$\frac{\mathrm{d[K]_{ap}}^{\mathrm{n,k}}}{\mathrm{dt}} = \frac{1}{\mathrm{a}}\left(\frac{\mathrm{V_{ap}}}{\mathrm{V_{cell}}}\right)^{-1} (\mathrm{j_{IRKC}}^{\mathrm{n,k}} + \mathrm{j_{ORKC}}^{\mathrm{n,k}} + \mathrm{j_{Ant}}^{\mathrm{n,k}}) + \mathrm{j_K}^{\mathrm{n,k/n-1,k}} + \mathrm{j_K}^{\mathrm{n,k/n+1,k}} + \\ + \mathrm{j_K}^{\mathrm{n,k/n,k-1}} + \mathrm{j_K}^{\mathrm{n,k/n,k+1}} \quad \text{(S33)},$$

$$\frac{\mathrm{d[K^+]_{cyt}}^{\mathrm{n,k}}}{\mathrm{dt}} = -\frac{1}{\mathrm{a}}\left(\frac{\mathrm{V_{cyt}}}{\mathrm{V_{cell}}}\right)^{-1} (\mathrm{j_{IRKC}}^{\mathrm{n,k}} + \mathrm{j_{ORKC}}^{\mathrm{n,k}} + \mathrm{j_{Ant}}^{\mathrm{n,k}}) \quad \text{(S34)},$$

$$\frac{\mathrm{d[H]_{ap}}^{\mathrm{n,k}}}{\mathrm{dt}} = \frac{1}{\mathrm{a}}\left(\frac{\mathrm{V_{ap}}}{\mathrm{V_{cell}}}\right)^{-1} (\mathrm{j_P}^{\mathrm{n,k}} - \mathrm{j_{Ant}}^{\mathrm{n,k}}) + \mathrm{j_H}^{\mathrm{n,k/n-1,k}} + \mathrm{j_H}^{\mathrm{n,k/n+1,k}} + \\ + \mathrm{j_H}^{\mathrm{n,k/n,k-1}} + \mathrm{j_H}^{\mathrm{n,k/n,k+1}} \quad \text{(S35)},$$

$$\frac{\mathrm{d[H]_{cyt}}^{\mathrm{n,k}}}{\mathrm{dt}} = -\frac{1}{\mathrm{a}}\left(\frac{\mathrm{V_{cyt}}}{\mathrm{V_{cell}}}\right)^{-1} (\mathrm{j_P}^{\mathrm{n,k}} - \mathrm{j_{Ant}}^{\mathrm{n,k}}) \quad \text{(S36)},$$

where $\frac{\mathbf{V_{cyt}}}{\mathbf{V_{cell}}}$ was ratio of the cytoplasmic volume to the total cell volume.

The model included description of buffer capacity of the apoplast for K^+ and H^+ and buffer capacity of the cytoplasm for H^+ [8-9]. Equation (S37) described buffer capacity of the cytoplasm:

$$[\mathrm{H^+}]_{\mathrm{cyt}} = \frac{\left([\mathrm{H}]_{\mathrm{cyt}} - \mathrm{B_{cyt}} - \mathrm{K_H}^{cyt}\right)}{2} + \frac{\sqrt{\left([\mathrm{H}]_{\mathrm{cyt}} - \mathrm{B_{cyt}} - \mathrm{K_H}^{cyt}\right)^2 + 4\mathrm{K_H}^{cyt}[\mathrm{H}]_{\mathrm{cyt}}}}{2} \quad \text{(S37)},$$

where B_{cyt} was total concentration of proton buffer (free and bonded) in the cytoplasm, K_H^{cyt} was the dissociation constant between the cytoplasmic buffer and H^+.

Equations (S38) and (S39) described buffer capacity of the apoplast [9]:

$$[H^+]_{ap} = \frac{-K_H{}^{ap}[H]_{ap}(B_{ap}-[H]_{ap}-[K]_{ap}-K_K{}^{ap})}{2(K_K{}^{ap})(B_{ap}-[H]_{ap})} + \quad (S38),$$

$$+\frac{\sqrt{\left(K_H{}^{ap}[H]_{ap}(B_{ap}-[H]_{ap}-[K]_{ap}-K_K{}^{ap})\right)^2+4K_K{}^{ap}(K_H{}^{ap})^2([H]_{ap})^2(B_{ap}-[H]_{ap})}}{2K_K{}^{ap}(B_{ap}-[H]_{ap})}$$

$$[K^+]_{ap} = \frac{K_K{}^{ap}[H^+]_{ap}[K]_{ap}}{K_H{}^{ap}[H]_{ap}+K_K{}^{ap}[H^+]_{ap}} \quad (S39),$$

where B_{apt} was total concentration of proton buffer (free and bonded) in the apoplast, $K_H{}^{ap}$ and $K_K{}^{ap}$ were the dissociation constants between the apoplastic buffer and H^+ and between the apoplastic buffer and K^+.

Concentration of H^+ in stroma ($[H^+]_{str}$) was assumed as constant.

8. Description of changes in ATP concentration

Stationary concentration of ATP ([ATP]), which was calculated per unit of the leaf volume, was described by Equation (40):

$$[ATP] = \frac{\beta(R_d + \alpha \cdot \min(W_c, W_j))}{\beta\left(R_d + \alpha \cdot \min(W_c, W_j)\right) + k_{cons}} ATP_\Sigma \quad (S40),$$

where $\beta = \frac{S_{leaf}}{V_{leaf}} \frac{5}{ATP_\Sigma - [ATP]_{dark}}$ was additional parameter, $\frac{S_{leaf}}{V_{leaf}}$ was ratio of the leaf area to the leaf volume, ATP_Σ and $[ATP]_{dark}$ were total concentration of ATP and ADP and concentration of ATP under dark conditions (without photosynthetic processes), α was portion of the CO_2 assimilation rate which was used for the ATP synthesis, and k_{cons} was effective velocity constant of all processes of the ATP consumption (this parameter was calculated on basis of only Rd under dark conditions).

9. Parameterization of the model

Table S1 shows main parameters of the model which were used in the work. Initial values of concentrations of H^+ and K^+ (and their total concentrations) in the apoplast and cytoplasm and concentrations of CO_2 in the apoplast, cytoplasm, and stroma of chloroplasts, which were variables of our model, are also shown in this table. It should be noted that initial concentrations of HCO_3^- and initial summary concentrations of CO_2 and HCO_3^- were calculated on basis of initial concentrations of CO_2 and equation (S17).

Table S1. Parameters used in the two-dimensional photosynthetic model

Parameters	*Values*	*Units*	*Sources for calculation of values*
	Photosynthetic CO_2 assimilation and photorespiration		
**K_C**	260	ppm	[1]
**K_O**	179000	ppm	[1]
**V_{max}**	80	μmol m^{-2} s^{-1}	[1]
**$Γ^*$**	38.6	ppm	[1]
**$[O_2]$**	200000	ppm	[1]
**J_{max}**	160	μmol m^{-2} s^{-1}	[1]
**abs**	0.85		[1]
**f**	0.15		[1]
**θ**	0.7		[1]
**R_d**	1	μmol m^{-2} s^{-1}	[1]

	Stomata and transmembrane CO_2 fluxes		
**$[CO_2]_{out}$**	360	ppm	Assumed
**g_s^0 (model elements with both mesophyll cell and stomata)**	0.576 (basic) or 0.207 (decreased)	mol m^{-2} s^{-1}	The basic g_s^0 was calculated as g_s·9, where $g_s = 0.064$ mol m^{-2} s^{-1} (the current experiment). The decreased g_s^0 was calculated as 0.576·9/25
**g_s^0 (model elements without stomata)**	0	mol m^{-2} s^{-1}	Assumed
**g_m**	0.1	mol m^{-2} s^{-1}	[12]
$\frac{S_{PM}}{S_{Chl}}$	0.495		[4-5]
	Lateral CO_2 and HCO_3^- fluxes		
**a**	10^{-3}	dm	[6]
**D_{CO2}**	$1.83 \cdot 10^{-7}$	dm^2 s^{-1}	[5]
**D_{HCO3}**	$0.95 \cdot 10^{-7}$	dm^2 s^{-1}	[5]
$\frac{V_{ap}}{V_{cell}}$	0.1		[11]
	Changes in CO_2 and HCO_3^- concentrations		
$\frac{S_{leaf}}{V_{ap}}$	5500	dm^{-1}	[11, 13]
$\frac{S_{leaf}}{V_{cyt}}$	13750	dm^{-1}	[3, 11, 13]
$\frac{S_{leaf}}{V_{str}}$	6790	dm^{-1}	[3, 11, 13]
**pK**	6.35		[7]
	Description of transmembrane H^+ and K^+ fluxes and membrane potential		
	H^+-ATPase		
**k_1**	0.045	s^{-1}	[9]
**k_2**	$2.58 \cdot 10^{-5}$	s^{-1}	[9]
**T**	296	K	[8]
**F**	96500	C mol^{-1}	[8]
**R**	8.31	J mol^{-1} K^{-1}	[8]
**G_{ATP}**	-50000	J	[8]
**K_{ATP}**	$186 \cdot 10^{-6}$	M	[14]
**K_{cyt}**	5.676		[3]
**A_{BL0}**	0.566		[14]
**K_{BL}**	6.55	μmol m^{-2}s^{-1}	[14]
	Inwardly and outwardly rectifying K^+ channels		
**P_{max}^{IRKC}**	$2.9 \cdot 10^{-7}$	dm s^{-1}	[9]
**P_{max}^{ORKC}**	$2.9 \cdot 10^{-7}$	dm s^{-1}	[9]
**u_{IRKC}**	-7.4		[8]
**u_{ORKC}**	-2.53		[8]
**c_{IRKC}**	1.1		[8]
**c_{ORKC}**	1.13		[8]

	K^+/H^+-antiporter		
k_{ant}	0.015	M^{-1} s^{-1}	[8]

Description of lateral H^+ and K^+ fluxes

D_H	$7.8 \cdot 10^{-7}$	dm^2 s^{-1}	[6]
D_K	$1.96 \cdot 10^{-7}$	dm^2 s^{-1}	[6]

Description of changes in K^+ and H^+ concentrations

$\frac{V_{cyt}}{V_{cell}}$	0.04		[3]
B_{cyt}	0.2	M	[8]
K_H^{cyt}	10^{-6}	M	[8]
B_{ap}	0.083	M	[8]
K_H^{ap}	10^{-6}	M	[8]
K_K^{ap}	10^{-4}	M	[8]
$[H^+]_{str}$	$3.16 \cdot 10^{-8}$	M	[15]

Description of changes in ATP concentration

α	0.2		Assumed
ATP_Σ	$0.132 \cdot 10^{-3}$	M	[16]
$[ATP]_{dark}$	$0.065 \cdot 10^{-3}$	M	[16]
k_{cons}	0.3846	s^{-1}	Assumed
$\frac{S_{leaf}}{V_{leaf}}$	5000	m^2 m^{-3}	[13]

Initial values of main variables

$[CO_2]_{ap}$	360	ppm	Assumed
$[CO_2]_{cyt}$	360	ppm	Assumed
$[CO_2]_{str}$	360	ppm	Assumed
$[K]_{ap}$ / $[K^+]_{ap}$	$8.2 \cdot 10^{-2}$ / $3.5 \cdot 10^{-3}$	M	[8]
$[K^+]_{cyt}$	$1.4 \cdot 10^{-1}$	M	[17]
$[H]_{ap}$ / $[H^+]_{ap}$	$2 \cdot 10^{-3}$ / 10^{-6}	M	[8]
$[H]_{cyt}$ / $[H^+]_{cyt}$	$1.5 \cdot 10^{-2}$ / $7 \cdot 10^{-8}$	M	[8]

References for Supplementary Materials

1. von Caemmerer, S.; Farquhar, G.; Berry, J. Biochemical model of C_3 photosynthesis, In *Photosynthesis in silico. Advances in Photosynthesis and Respiration.* Laisk, A., Nedbal, L., Govindjee, Eds. Springer, Dordrecht, Germany, 2009; Volume 29, pp. 209-230.
2. Bernacchi, C.J.; Rosenthal, D.M.; Pimentel, C.; Long, S.P.; Farquhar, G.D. Modeling the temperature dependence of C_3. In *Photosynthesis in silico. Advances in Photosynthesis and Respiration*. Laisk, A., Nedbal, L., Govindjee Eds. Springer, Dordrecht, Germany, 2009; Volume 29, pp. 231-246.
3. Winter, H.; Robinson, D.G.; Heldt, H.W. Subcellular volumes and metabolite concentrations in spinach leaves. *Planta*. **1994**, *193*, 530-535.
4. Evans, J.R.; Kaldenhoff, R.; Genty, B.; Terashima, I. Resistances along the CO_2 diffusion pathway inside leaves. *J. Exp. Bot.* **2009**, *60*, 2235–2248.
5. Tholen, D.; Zhu, X.-G. The mechanistic basis of internal conductance: a theoretical analysis of mesophyll cell photosynthesis and CO_2 diffusion. *Plant Physiol.* **2011**, *156*, 90-105.
6. Sukhov, V.; Nerush, V.; Orlova, L.; Vodeneev, V. Simulation of action potential propagation in plants. *J. Theor. Biol.* **2011**, *291*, 47-55.

7. Sukhova, E.M.; Sukhov, V.S. Dependence of the CO_2 uptake in a plant cell on the plasma membrane H^+-ATPase activity: theoretical analysis. *Biochem. Moscow Suppl. Ser. A.* **2018**, *12*, 146-159.
8. Sukhov, V.; Vodeneev, V. A mathematical model of action potential in cells of vascular plants. *J. Membr. Biol.* **2009**, *232*, 59–67.
9. Sukhova, E.; Ratnitsyna, D.; Sukhov, V. Stochastic spatial heterogeneity in activities of H^+-ATP-ases in electrically connected plant cells decreases threshold for cooling-induced electrical responses. *Int. J. Mol. Sci.* **2021**, *22*, 8254.
10. Sukhova, E.; Akinchits, E.; Sukhov, V. Mathematical models of electrical activity in plants. *J. Membr. Biol.* **2017**, *250*, 407-423.
11. Gradmann, D. Impact of apoplast volume on ionic relations in plant cells. *J. Membr. Biol.* **2001**, *184*, 61–69.
12. Flexas, J.; Barbour, M.M.; Brendel, O.; Cabrera, H.M.; Carriquí, M.; Díaz-Espejo, A.; Douthe, C.; Dreyer, E.; Ferrio, J.P.; Gago, J.; Gallé, A.; Galmés, J.; Kodama, N.; Medrano, H.; Niinemets, Ü.; Peguero-Pina, J.J.; Pou, A.; Ribas-Carbó, M.; Tomás, M.; Tosens, T.; Warren, C.R. Mesophyll diffusion conductance to CO_2: an unappreciated central player in photosynthesis. *Plant Sci.* **2012**, *193-194*, 70-84.
13. Day, T.A.; Vogelmann, T.C. Alterations in photosynthesis and pigment distributions in pea leaves following UV-B exposure. *Physiol. Plant*. **1995**, *94*, 433-440.
14. Kinoshita, T.; Shimazaki, K. Blue light activates the plasma membrane H^+-ATPase by phosphorylation of the C-terminus in stomatal guard cells. *EMBO J.* **1999**, *18*, 5548–5558.
15. Antal, T.K.; Kovalenko, I.B.; Rubin, A.B.; Tyystjärvi, E. Photosynthesis-related quantities for education and modeling. *Photosynth Res*. **2013**, *117*, 1-30.
16. Roeske C.A.; Chollet R. Role of metabolites in the reversible light activation of pyruvate, orthophosphate dikinase in *Zea mays* mesophyll cells *in Vivo*. *Plant Physiol*. **1989**, *90*, 330-337.
17. Wang, Y.; Wu, W.H. Plant sensing and signaling in response to K^+-deficiency. *Mol. Plant.* **2010**, *3*, 280-287.
